# The Role of Digital Fluorescence in Acne Vulgaris: Correlation of Ultraviolet Red Fluorescence with the Severity of Acne Vulgaris

**DOI:** 10.1155/2019/4702423

**Published:** 2019-12-28

**Authors:** Imam Budi Putra, Nelva K. Jusuf, Nani Kumala Dewi

**Affiliations:** Department of Dermatology and Venereology, Faculty of Medicine, Universitas Sumatera Utara, Medan, Indonesia

## Abstract

**Background:**

Colonization of *Propionibacterium acnes (P. acnes)* and increased sebum production play important roles in the pathogenesis of acne vulgaris. Severity of acne vulgaris correlates with the lesion counts both noninflammatory and inflammatory. Digital fluorescence has been found useful in pathogenesis investigation and treatment evaluation. Ultraviolet-induced red fluorescence (UVRF) was found to be correlated with sebum and porphyrin production that can be synthesized by *P. acnes*. Therefore, UVRF assessment could be useful for the evaluation of the degree and extent of acne vulgaris.

**Objective:**

To evaluate the correlation of UVRF with the severity of acne vulgaris using the digital fluorescence tool.

**Methods:**

Forty-five patients were diagnosed with mild-to-severe acne vulgaris according to Lehmann classification. Lesion counts both noninflammatory and inflammatory and UVRF assessment using Visiopor PP34 camera were done to all participants in 5 divided facial areas (forehead, nose, right and left cheeks, and chin). Clinical assessment for each patient was done by 3 dermatologists. Determination of correlation between UVRF with acne lesion counts was done using Pearson test and with acne severity using Spearman test.

**Results:**

From 45 participants, majority had moderate acne (64.4%), followed by severe (24.5%) and mild acne (11.1%). Mean number of UVRF spots was 39.98 ± 11.45 and percentage area covered with UVRF was 4.39 ± 1.72. There was no correlation found between acne lesion counts, including noninflammatory and inflammatory with the number and percentage area covered with UVRF spots. Severity grade of acne vulgaris was found to be uncorrelated with the number of UVRF spots (*r* = 0.27, *p*=0.073) and percentage area covered with UVRF spots (*r* = 0.173, *p*=0.256).

**Conclusion:**

The severity of acne vulgaris has no correlation with spot counts and percentage area covered with UVRF. Digital fluorescence might be helpful in investigating further of the interrelated pathogenesis factors of acne.

## 1. Introduction

Acne vulgaris is a chronic pilosebaceous inflammatory disorder with multiple etiology and pathogenesis factors [1]. Increased sebum production, follicular hyperkeratinization, inflammation, and colonization of *Propionibacterium (P. acnes) acnes* are the four major pathophysiologies [2]. Hormonal stimulation such as androgen induces sebaceous gland production and follicular keratinocyte proliferation. In consequence, follicular impactions lead to development of microcomedones. With increase of sebum and corneocytes accumulation, these initially invisible lesions may progress into clinically evident comedones. They become a preferred microenvironment for colonization of cutaneous bacteria, especially *P. acnes*. These anaerobe Gram-negative bacteria also play a peculiar role in promoting inflammation with proinflammatory mediator-releasing activity such as lipase, protease, hyaluronidase, and chemotactic factors, leading to development of inflammatory acne lesions such as papules, pustules, and nodules/cysts [3–5].

Furthermore, follicular colonization of *P. acnes* in addition to accumulation of sebum was found to produce porphyrins in the form of coproporphyrin III and protoporphyrin IX [6]. They are known as *Propionibacteria* endogenous metabolites that are able to promote perifollicular inflammation with stimulation in cytotoxic squalene oxide production and release of keratinocyte-derived IL-8 [7, 8]. These proinflammatory metabolites are native fluorophores and strongly fluorescent under ultraviolet A (UVA), which appear as follicular orange-red fluorescence and known as ultraviolet-induced red fluorescence (UVRF) [9, 10]. Some studies have reported the use of photographic techniques in the evaluation of the correlation between *P. acnes* colonization and UVRF. The intensity of follicular fluorescence and the extent of facial involvement have positive correlation with the density of *P. acnes* [11, 12]. Reduction in porphyrin concentration and UVRF with acne vulgaris treatment was found proportionate with the clinical improvement. Therefore, the determination of UVRF could be useful for the evaluation of the degree and extent of acne vulgaris [6, 12, 13]. The aim of this study is to evaluate the correlation of UVRF with the severity of acne vulgaris, using a digital fluorescence tool.

## 2. Methods

Forty-five patients diagnosed with mild-to-severe acne vulgaris according to Lehmann classification, aged 18–35 years old, were included after giving their written informed consent. All the patients who had inflammatory and noninflammatory acne lesions on the face, not in pregnancy or lactation, did not take any topical or oral medication for at least 1 month prior to the study. This study was done under approval of the Ethics Committee of Universitas Sumatera Utara General Hospital. Clinical assessment was done by counting inflammatory (papule, pustule, nodule, and cyst) and noninflammatory (open and closed comedones) acne lesions on 5 divided areas of the face: forehead, nose, right cheek, left cheek, and chin. The severity of disease was rated in mild, moderate, and severe scale based on the total number of inflammatory and noninflammatory acne lesions. The clinical assessment was done by 3 certified dermatologists for each patient.

The UVRF was determined by Visiopor PP 34 camera (Courage + Khazaka, Cologne, Germany), which uses a specific UVA light (375 nm) with a measured area of 6 × 8 mm. The porphyrins are visible as fluorescent orange-red spots in the pores, which indicate the presence of *P. acnes* bacteria living within and on the surface of the follicular impactions or comedones. The parameters analyzed were the number and percentage of the area covered by orange-red spots. The yellow color spots in the images were excluded from the analysis. The measured area was divided into 5: forehead, nose, right and left cheeks, and chin, and then the average of fluorescence parameters was calculated.

## 3. Statistical Analysis

Normality distribution test using Saphiro–Wilk test was done for all set of data. The correlation between the number of inflammatory, noninflammatory, and total of both acne lesions with UVRF parameters were evaluated using Pearson correlation test. Relationship between severity grade of acne vulgaris with the number and percentage of area covered by orange-red fluorescence spots were determined using the Spearman correlation test. *p* < 0.05 was considered statistically significant.

## 4. Results

Among 45 study participants (Table 1), 62.2% were female, and majority were in the age group of 18–25 years old (84.5%). Most of the participants had moderate acne (64.4%), followed by severe (24.5%) and mild acne (11.1%). Mean number of total acne vulgaris lesion was 110.67 ± 49.56, including noninflammatory (88.22 ± 41.94) and inflammatory lesions (22.44 ± 15.83). From all the participants, the average number of UVRF spots found in 5 divided facial areas ranged from 12.2 to 67.6 with mean 39.98 ± 11.45. The average of percentage area covered with UVRF spot was 4.39 ± 1.72 that ranged from 1.27 to 10.78 (Table 2).

In correlation statistical analysis, there was no correlation found between acne lesion counts, including noninflammatory and inflammatory with the number and percentage area covered with UVRF spots (Table 3). Furthermore, severity grade of acne vulgaris was found to be uncorrelated with the number of UVRF spots (*r* = 0.27, *p*=0.073) and percentage area covered with UVRF spots (*r* = 0.173, *p*=0.256) as presented in Table 4.

## 5. Discussion

This study confirmed that there is no correlation between the severity grades of acne vulgaris with UVRF. Lehmann classification was used to determine acne severity grade in this study, which evaluates noninflammatory, inflammatory, and total number of lesions. In results, severity grade correlates with the number of lesions both inflammatory and noninflammatory. A previous study by Xu et al. also found that UVRF of hair follicles in acne vulgaris patients does not correlate with the inflammation severity. However, this study only determined the severity based on inflamed lesions using chromatologic evaluation, not by acne grading severity as in our study [14].

Furthermore, the type of lesions, either noninflammatory or inflammatory are also found to be uncorrelated with the UVRF in our study. In contrast, Kim et al. reported that noninflammatory lesion correlates significantly with red fluorescence (UVRF), but inflammatory lesion with green fluorescence [15]. A previous study by Okoro et al. has shown positive correlation between acne lesion counts and facial red fluorescence on the cheeks and chin (*U* zone) but not *T* zone (forehead and nose) [16]. This variation of results might be due to the subgroup analysis of different areas in the prior study and might give more insight of the significance of UVRF on acne lesions in each facial area. According to Lucchina et al. inflammatory lesions were found to fluoresce less and more inconsistent than open comedones and follicles [12]. In inflammatory lesions, colonization of *P. acnes* usually found fewer due to its anaerobic nature that tend to avoid higher oxygen tension in inflammation [17]. In terms of noninflammatory lesions, only open comedones give yellowish-white fluorescence under UVA illumination due to keratin plugs, but closed comedones do not fluoresce [12]. These findings might explain the absence of correlation between acne vulgaris lesions both inflammatory and noninflammatory with UVRF.

UVRF is known to be emitted by *Propionibacterium acnes* indicating the presence of porphyrin (coproporphyrin III and protoporphyrin IX) [18]. As the metabolite of anaerobic Gram-negative bacteria, majority of the orange-red spots are usually found in the follicle. Initially, this follicular orange-red fluorescence is thought to correspond only with colonization of *P. acnes* [11]. The intensity of UVRF was found to be proportional with the quantity of *P. acnes* and correlated directly with their reduction and clinical improvement after treatment [10, 12, 13]. However, recent studies have shown sebum level and other microorganisms might play a significant role in resulting the emission of this fluorescence [16, 19, 20].

Youn et al. reported that sebum amount correspond more closely with UVRF rather than the presence of *P. acnes* [19]. Okoro et al. also confirmed that facial red fluorescence has positive correlation with sebum level especially at the cheeks and chin (*U* zone). At the *T* zone, high density of sebaceous glands results in abundant fluorescence level. In contrast, the *U* zone emits lower basic fluorescence that allows better distribution of values among normal and acne-involved skin and thus more significant correlations [16]. Furthermore, Xu et al. found that the colors of lesional fluorescence are mainly nonred (86.8%), while nonlesional follicles are mostly red (*x*^2^ = 222.87, *p* < 0.01). The fluorescence of corneum plugs isolated from acne vulgaris patients revealed only 11% is mainly red. These results suggest the possibility of other microorganisms that might play a role in acne vulgaris pathogenesis [20]. Further study revealed that *Staphylococcus epidermidis* isolated from follicles of acne vulgaris patients emits UVRF when anaerobically cultured and then exposed to air [21]. The fluorescence spectrum is identical with coproporphyrin III. Since it can convert 5*α*-aminolevulinic acid into porphyrin, *S. epidermidis* might have potential to produce porphyrin [22]. Put into these considerations, the pathogenesis behind UVRF might not be as simple as involving only *P. acnes* and porphyrin, but also other etiologic factors of acne vulgaris such as sebum production and other microorganisms. Therefore, digital fluorescence might be useful to investigate further the association between each pathogenesis of acne vulgaris especially colonization of microorganism and sebum production.

As a multifactorial etiologic condition, severity of acne vulgaris is associated not only with the presence of *P. acnes* and sebum production but also inflammation and proliferation of follicular keratin [5]. Therefore, in this study, we found no correlation between acne severity grade with UVRF, which is known to indicate colonization of *P. acnes* and sebum level. Although it does not correlate with the severity of acne vulgaris, UVRF examination has been used in several studies to evaluate the efficacy of treatments [10, 12, 13]. Borelli et al. reported significant reduction in porphyrin fraction of patients with isotretinoin treatment in just 2 months after starting therapy and was associated with the clinical improvement [10]. Lucchina et al. also reported the use of UVRF in evaluating clindamycin treatment on acne vulgaris patients which showed significant reduction in fluorescence after therapy [12]. Even Pagnoni et al. suggested UVRF as an indicator of suppressive effect of benzoyl peroxide on *P. acnes* [13]. However, further studies are required to confirm mechanism behind the reduction effect of treatment on UVRF, whether due to porphyrin depletion, inhibition activity against *P. acnes* and other microorganism, or decrease in sebum production.

## 6. Conclusion

As a multiple etiologic condition, the severity of acne vulgaris has no correlation with spot counts and percentage area covered with UVRF. Recent studies have shown the role of sebum production and colonization of *P. acnes* and *S. epidermidis* in the development of this follicular fluorescence. The role of digital fluorescence in the clinical and research evaluation of acne is still evolving, and further study may or may not prove it to be a useful tool moving forward.

## Figures and Tables

**Table 1:** Demograhic data.

Data	*n* (%)
Total subjects	45 (100)

Sex	
Male	17 (37.8)
Female	28 (62.2)

Age (years old)	
18–25	38 (84.5)
26–30	6 (13.3)
>30	1 (2.2)

Severity	
Mild	5 (11.1)
Moderate	29 (64.4)
Severe	11 (24.5)

**Table 2:** Characteristic of acne vulgaris lesions and UVRF parameters.

Parameter	Minimum	Maximum	Median	Mean ± SD
Acne lesion counts				
Noninflammatory	20	192	88	88.22 ± 41.94
Inflammatory	3	66	17	22.44 ± 15.83
Total	28	235	118	110.67 ± 49.56

UVRF spots				
Number	12.2	67.6	41	39.98 ± 11.45
Percentage of area covered	1.27	10.78	4.36	4.39 ± 1.72

**Table 3:** Correlation between the type of acne vulgaris lesions with UVRF parameters.

Acne vulgaris lesion		UVRF spots	
		Number	Percentage of area covered
Noninflammatory lesion	*r*	0.206	0.267
	*p*	0.174	0.077

Inflammatory lesion	*r*	0.013	−0.095
	*p*	0.932	0.536

Total lesion	*r*	0.178	0.194
	*p*	0.243	0.203

**Table 4:** Correlation between severity grades of acne vulgaris with UVRF parameters.

		UVRF spots	
		Number	Percentage of area covered
Severity grades of acne vulgaris	*r*	0.27	0.173
	*p*	0.073	0.256

## Data Availability

All the data used to support the findings of this study are available from the corresponding author upon request.
